# Research priority seven: What is the role of occupational therapy in supporting self-management? Developing an occupation-centred lens for research and practice

**DOI:** 10.1177/03080226231197312

**Published:** 2023-08-30

**Authors:** Niamh Kinsella, Julie King

**Affiliations:** Queen Margaret University, Edinburgh, UK

The seventh research priority identified in the James Lind Alliance (JLA) Priority Setting Partnership is: ‘*what is the role of occupational therapy in supporting self-management?*’ The JLA identified that existing research related to self-management focuses on demonstrating the effectiveness of specific interventions intended to enhance peoples’ ability to manage their own health. The JLA listened to service users and occupational therapists who suggested that this focus on intervention effectiveness does not sufficiently explain the role of the occupational therapist in supporting self-management. This limitation in knowledge can result in practice that is not aligned with the philosophy of the profession and has the potential to overlook the desired occupation-centred outcomes of self-management.

Corbin and Strauss’ (1988) tripartite model of self-management is routinely used to define self-management practices in occupational therapy practice and research. Corbin and Strauss (1988) referred to self-management as the ‘medical, emotional and role management’ work a person does when living with a long-term condition. The model assumed that people can learn to take responsibility for their health by independently managing medication, the emotional burden of the condition, and changing lifestyle behaviours and roles. Though frequently referred to and relied upon to underpin practice and research in occupational therapy (Packer, 2013), this model and its assumptions are not derived from an occupation-centred discipline and thus, the centrality of occupation in associated self-management practice is often limited. This history offers some insight into the reason for the uncertainty and ambiguity about the professions’ role in self-management.

It has been proposed that the self-management agenda in healthcare more generally emerged as a response to healthcare needs of a growing population with long-term, chronic conditions in the United Kingdom (UK). This led to a call for public health system action in the UK and internationally. In the UK, this agenda was realised through establishment of the National Health Service’s Expert Patient Programme in 2002. The political agenda here perhaps reflected the idea that workforce resources could be managed through implementation of particular approaches to healthcare such as education about medication use and conditions, thus minimising the burden of long-term conditions on healthcare systems. This is reflected in research that aims to make associations between self-management practices and cost-effectiveness or reduced healthcare service utilisation (Barker et al., 2018).

The origins of the self-management movement in occupational therapy are more difficult to identify, with reference to self-management of people with chronic conditions made in the 1980s, at the same time as the emergence of theories and definitions of self-management. Despite the origins of self-management ideas and practices occurring beyond the occupational therapy profession, self-management started to appear as a politically driven expectation of occupational therapists in healthcare strategy and practice guidelines in the UK in the 2000s, with occupational therapists also involved in delivery of the Expert Patient Programmes. Occupational therapists were often viewed as well placed to do self-management with people with long-term conditions because of the alignment of professional principles with the idea of supporting people to be independent. The challenge faced by occupational therapists in this landscape has been the emergence of tensions between the professionally generic, politically driven self-management practices and the requirement to apply an occupational lens to these self-management practices. Thus, agreeing a professional stance on what self-management means for our profession is fundamental, alongside an effort to critically understand the origins and purpose of the agenda for the profession.

In adopting an occupational lens, it is frequently proposed that the core tenet of independence is the feature of the profession that aligns most clearly with the self-management agenda. The assumption here is that occupational therapists are well positioned to enable independent management of long-term conditions. This assumption can be contrasted with the core professional idea of occupation-centredness, defined by Fisher (2013), that occupational therapists should be concerned with achieving health through understanding and use of occupation, whether this be independent engagement in occupation or not. In other words, the value of independence has become somewhat of a red herring (distraction from the real issue) for occupational therapists in the context of self-management, with both practice and research activities in this area centring on the application of biomedical and behavioural sciences to peoples’ lives to achieve independent living.

Some examples of developments towards an occupation-centred perspective are offered in contemporary research, with research proposed to explore the role of occupation in managing long-term conditions (Gavin et al., 2022). However, this acknowledgement of the centrality of occupation has not been translated to occupation-based or occupation-focused practice thus far, with existing research focusing on the effectiveness of self-management processes in changing biomedical outcomes such as pain, fatigue and depression. Fisher’s (2013) occupation-related taxonomy challenges these approaches to practice and research, suggesting that occupation should be the process of change and outcome of interest in research and practice. An example is as follows:

Jo is hoping to remain in work to retirement age as she values it for emotional support and financial stability. She is currently experiencing a disruption to her performance of this occupation, particularly in maintaining routine and stamina^1^ in her working day, resulting in her taking several days sick leave. She refers herself to an occupational therapist who starts a conversation about what has changed in her life and observes her carrying out a work-related activity. Jo identifies that beginning work at her usual time is more effortful than normal, causing her to be too tired by lunch time to complete the work she is expected to do. The occupational therapist guides the intervention by referring to information and tools to support the management of fatigue and adjusting them to make them accessible for her, and by supporting a conversation about reasonable working adjustments with her employer. Over the following month, her fatigue does not subside. However, she reports improved work performance, being able to complete the work she is expected to do, giving her hope.

As this example indicates, the role of the occupational therapist is in incorporating adjusted self-management practices into an occupation that is prioritised in collaboration with a person. In a decontextualised self-management approach, the focus would have started on the condition, for example, multiple sclerosis, and the intervention being delivery of generalised information about condition-specific symptom management.

Self-management practice should be a dynamic, contextualised process, guided by peoples’ experience of occupation at any given moment. The contextualisation of self-management processes to occupational therapy can be supported by existing, occupation-centred practice process frameworks, such as *Occupational Therapy and Complexity: Defining and Describing Practice* (Pentland et al., 2018). Such frameworks outline the role of education as a core component of the therapeutic process, akin to any self-management process, but also offer a way of thinking about self-management practices *in relation to* peoples’ occupations. They also offer a guide to the educational approaches that may be chosen, recognising that education for self-management takes many forms, depending on peoples’ preferences and abilities. This perspective of self-management also addresses the concern raised by Hinder and Greenhalgh (2012) that engagement in, and success of, self-management practices are dependent on the contextual conditions in which the self-management activity occurs, such as financial position, culture and psychological state. For instance, the practice of fatigue management may not have been possible in the example if this had not been negotiated with an employer or if the self-management practice was not applied to an occupation that was identified as health-giving. In an occupation-centred approach, the occupational therapist would consider the contextual conditions that might limit application of the self-management practice.

If the JLA question is to be addressed, the dynamic, contextualised processes described here will need to be researched in realistic or naturalistic ways. It is inherently more challenging to research and demonstrate outcomes in this way as the therapeutic process will look different in each instance of practice. However, this work is fundamental in establishing the contextual variables that impact on the use of self-management activities as an approach to enhancing occupational performance and engagement. We must be brave here to embrace this research, using our occupation-centred frameworks and understanding of the complexity to support self-management that delivers occupation-centred outcomes. This also means a ‘letting go’ of reliance on traditional approaches to self-management practice, which are often applied in isolation of the therapeutic process, as informed by policy and as required to demonstrate outcomes in experimental research.

Ultimately, the need to demonstrate the role of the occupational therapist is derived from the requirement to continue to fund and justify practice, which can be grounded in profession-specific measures of occupational performance and engagement. Evidence is needed to demonstrate the effectiveness of occupation-centred self-management practices in enhancing occupational performance and engagement over time. This could clearly define the role and agenda of the profession in self-management practice and policy.

## Notes

1. Health condition is not shared here to support establishment of occupation-centredness in the case.
